# Reduced pain and discomfort after surgical repair of inguinal hernia in infants: secondary outcome analysis of the randomized controlled HERNIIA-trial

**DOI:** 10.1093/bjs/znag064

**Published:** 2026-06-20

**Authors:** Lore E de Vreeze, Sanne C Maat, Johannes R Anema, Robertine van Baren, Jasper V Been, Mart H M Bender, Hanneke M van Dongen, Hester R Langeveld-Benders, Ruben G J Visschers, Jos W R Twisk, Gerda W Zijp, Ernest L W van Heurn, Joep P M Derikx

**Affiliations:** Department of Pediatric Surgery, Emma Children's Hospital, Amsterdam UMC, Amsterdam, The Netherlands; Amsterdam Gastroenterology Endocrinology Metabolism, Amsterdam UMC Locatie AMC, Amsterdam, The Netherlands; Amsterdam Reproduction and Development Research Institute, Amsterdam UMC Locatie AMC, Amsterdam, The Netherlands; Department of Pediatric Surgery, Emma Children's Hospital, Amsterdam UMC, Amsterdam, The Netherlands; Amsterdam Gastroenterology Endocrinology Metabolism, Amsterdam UMC Locatie AMC, Amsterdam, The Netherlands; Amsterdam Reproduction and Development Research Institute, Amsterdam UMC Locatie AMC, Amsterdam, The Netherlands; Department of Public and Occupational Health, and the Amsterdam Public Health Research Institute, Amsterdam UMC, Vrije Universiteit Amsterdam, Amsterdam, The Netherlands; Department of Pediatric Surgery, Beatrix Children’s Hospital, University Medical Center Groningen, University of Groningen, Groningen, The Netherlands; Division of Neonatology, Department of Neonatal and Pediatric Intensive Care, Erasmus MC Sophia Children's Hospital, University Medical Center Rotterdam, Rotterdam, The Netherlands; Department of Public Health, Erasmus MC, University Medical Centre Rotterdam, Rotterdam, The Netherlands; Department of Obstetrics and Gynecology, Erasmus MC Sophia Children's Hospital, University Medical Center Rotterdam, Rotterdam, The Netherlands; Department of Surgery, Maxima Medical Center, Veldhoven, The Netherlands; Department of Health Sciences, Faculty of Science, Vrije Universiteit Amsterdam, Amsterdam Public Health Research Institute, Amsterdam, The Netherlands; Department of Pediatric Surgery, Sophia Children’s Hospital, Erasmus Medical Center, Rotterdam, The Netherlands; Department of Pediatric Surgery, MosaKids Children’s Hospital, Maastricht University Medical Center+ (MUMC+), Maastricht, The Netherlands; Department of Pediatric Surgery, European Consortium of Pediatric Surgery (Maastricht University Medical Center+, Uniklinik Aachen, Centre Hospitalier Chrétien Liège), Maastricht, The Netherlands; Department of Epidemiology and Data Science, and the Amsterdam Public Health Research Institute, Amsterdam UMC, Amsterdam, The Netherlands; Department of Pediatric Surgery, Juliana Children’s Hospital, Haga Hospital, Den Haag, The Netherlands; Department of Pediatric Surgery, Emma Children's Hospital, Amsterdam UMC, Amsterdam, The Netherlands; Amsterdam Gastroenterology Endocrinology Metabolism, Amsterdam UMC Locatie AMC, Amsterdam, The Netherlands; Amsterdam Reproduction and Development Research Institute, Amsterdam UMC Locatie AMC, Amsterdam, The Netherlands; Department of Pediatric Surgery, Emma Children's Hospital, Amsterdam UMC, Amsterdam, The Netherlands; Amsterdam Gastroenterology Endocrinology Metabolism, Amsterdam UMC Locatie AMC, Amsterdam, The Netherlands; Amsterdam Reproduction and Development Research Institute, Amsterdam UMC Locatie AMC, Amsterdam, The Netherlands

Dear Editor,

Inguinal hernia is the most common congenital abnormality requiring surgery in children. Surgical repair is warranted due to the high 10% incarceration rate^1^. Surgical timing is traditionally based on an equipoise between incarceration risk and concerns regarding anaesthetic safety^2^. Symptom burden plays no role in this decision-making, as infantile inguinal hernia is generally regarded as an asymptomatic condition. Parental observations however frequently suggest that symptoms, such as discomfort, inconsolable crying, and altered stool patterns often coincide with the presence of an inguinal hernia. Limited research has examined relief of these symptoms after repair^3^. Patient-reported outcome measures (PROMs) may aid in the objective assessment of symptom burden^4^. This study investigated the frequency of pain and discomfort in infants aged 0–6 months with unilateral inguinal hernia and changes in PROMs 4 weeks after repair, including risk factors for persistent postoperative pain and discomfort. Changes in colic, stomach ache, obstipation, and laxative use were also evaluated.

This is a planned secondary outcome analysis of data of the multicentre HERNIIA randomized controlled trial (ClinicalTrials.govNCT03623893), conducted in eight centres in the Netherlands and Belgium between April 2019 and May 2023^5^. A total of 402 infants aged 0–6 months underwent open unilateral inguinal hernia repair and parents completed proxy versions of the EQ-5D-5L and TAPQOL questionnaires preoperatively and 4 weeks after surgery. Full trial details are provided in the Supplementary material. Primary outcome of the present analysis was the presence of pain and discomfort. Secondary outcomes included colic, stomach ache, obstipation, and laxative use. Paired comparisons were analysed using McNemar’s test. Multivariable logistic regression was performed to identify risk factors for persistent pain and discomfort at 4 weeks. Methodological details and 1-year follow-up data are provided in the Supplementary material (*Table S2*).

Characteristics were similar in the two treatment groups of the trial (that is contralateral exploration *versus* control, *Table S1*) and questionnaires responders and non-responders (*Tables S1* and *S2*). Response rates were 60% preoperatively and 68% following surgery. Among infants with paired data, 137 of 200 (68.5%) experienced pain and discomfort before surgery. This proportion decreased to 48 (24.0%) 4 weeks after repair (*P* < 0.001) (*Table 1*). Colic was reported in 194 of 204 infants (95.1%) preoperatively and decreased to 173 (84.8%) following surgery (*P* < 0.001). Obstipation declined from 68 of 400 (17.0%) to 26 (6.5%) (*P* < 0.001), and laxative use from 38 of 402 (9.5%) to 22 (5.5%) (*P* = 0.030). Stomach ache did not change significantly (46.1% *versus* 41.2%; *P* = 0.250).

**Table 1:** Changes in reported symptoms before and after hernia repair

	Prior to surgery	4 weeks after surgery	
	*n*/*n* (%)	*n*/*n* (%)	P*
Pain and discomfort	137/200 (68.5)	48/200 (24.0)	<0.001
Stomach ache or abdominal pain	94/204 (46.1)	84/204 (41.2)	0.250
Colic	194/204 (95.1)	173/204 (84.8)	<0.001
Obstipation	68/400 (17.0)	26/400 (6.5)	<0.001
Laxative use	38/402 (9.5)	22/402 (5.5)	0.030

Median time between preoperative questionnaire completion and surgery was 2 days (i.q.r. 1–5). Median time between surgery and postoperative questionnaire completion was 33 days (i.q.r. 28–40). *McNemar’s test.

Multivariable analysis including hernia side, incarceration history, and hernial sac content showed that right-sided hernia was associated with persistent pain and discomfort at 4 weeks (*P* = 0.045, OR 1.898, 95% c.i.: 1.014 to 3.552; *Table S3*).

These findings suggest that symptoms (for example discomfort, cramps, obstipation) often attributed by clinicians to normal gastrointestinal maturation or changes in nutrition during infancy are potentially related to the inguinal hernia. The decrease in reported pain and discomfort, colic, and obstipation likely reflect the effect of the hernia repair, as this is the consistent event happening between the two proximate time points. However, a possible beneficial effect of time on these symptoms cannot be ruled out completely. Limitations entail proxy-reported outcomes, no comparison with a healthy control group, and obstipation not assessed using formal Rome IV criteria. Right-sided hernia was independently associated with persistent pain and discomfort; this finding should be interpreted cautiously given the modest statistical significance.

Overall, these findings emphasize the importance of recognizing potential symptomatic presentations of infantile inguinal hernia and counselling parents accordingly. Finding clinical equipoise between incarceration risk, symptom burden, and the potential anaesthetic and respiratory risks associated with surgery is key to optimizing outcomes for both infants and their families.

## Supplementary Material

znag064_Supplementary_Data

## Data Availability

This report does not contain patient-identifiable data. Data in this report are anonymized. The data that support the findings of this study are available from the corresponding author upon reasonable request.

## Category and registration

This manuscript presents a research letter based on secondary outcome data from the multicentre randomized controlled trial titled ‘The Hernia Exploration oR Not In Infants Analysis (HERNIIA)’ (ClinicalTrials.gov identifier: NCT03623893, study start 2019–04–17 and study completion 2023–05–15, published in the *International Journal of Surgery* (2026;112:3661–3668; doi:10.1097/JS9.0000000000003863).
