# Supplementary material for: Reduced pain and discomfort after surgical repair of inguinal hernia in infants: secondary outcome analysis of the randomized controlled HERNIIA-trial
Source: Br J Surg. 2026 Jun 20;113(7):znag064. doi: 10.1093/bjs/znag064 (PMC13325674; doi:10.1093/bjs/znag064)
Supplement: znag064_Supplementary_Data [file znag064_supplementary_data.docx]

**Reduced pain and discomfort after surgical repair of inguinal hernia in infants: secondary outcome analysis of the randomized controlled HERNIIA-trial**

**Lore E. de Vreeze^1,2,3^, Sanne C. Maat^1,2,3^, Johannes R. Anema^4^, Robertine van Baren^5^, Jasper V. Been^6,7,8^, Mart H.M. Bender^9^, Hanneke M. van Dongen^10^, Hester R. Langeveld-Benders^11^, Ruben G.J. Visschers^12, 13^, Jos W.R. Twisk^14^, Gerda W. Zijp^15^, Ernest L.W. van Heurn^1,2,3,^ Joep P.M. Derikx ^1,2,3^**

^1^ Department of Pediatric Surgery, Emma Children's Hospital, Amsterdam UMC, Amsterdam, the Netherlands

^2^ Amsterdam Gastroenterology Endocrinology Metabolism, Amsterdam UMC Locatie AMC, Amsterdam, The Netherlands

^3^ Amsterdam Reproduction and Development research institute, Amsterdam UMC Locatie AMC, Amsterdam, The Netherlands

^4^ Department of Public and Occupational Health, and the Amsterdam Public Health Research Institute, Amsterdam UMC, Vrije Universiteit Amsterdam, Amsterdam, the Netherlands.

^5^ Department of Pediatric Surgery, Beatrix Children’s Hospital, University Medical Center Groningen, University of Groningen, Groningen, the Netherlands

^6^ Division of Neonatology, Department of Neonatal and Pediatric Intensive Care, Erasmus MC Sophia Children's Hospital, University Medical Center Rotterdam, Rotterdam, the Netherlands

^7^ Department of Public Health, Erasmus MC, University Medical Centre Rotterdam, Rotterdam, the Netherlands

^8^ Department of Obstetrics and Gynecology, Erasmus MC Sophia Children's Hospital, University Medical Center Rotterdam, Rotterdam, the Netherlands

^9^ Department of Surgery, Maxima Medical Center, Veldhoven, the Netherlands

^10^ Department of Health Sciences, Faculty of Science, Vrije Universiteit Amsterdam, Amsterdam Public Health Research Institute, Amsterdam, the Netherlands

^11^ Department of Pediatric Surgery, Sophia Children’s Hospital, Erasmus Medical Center, Rotterdam, the Netherlands

^12^ Department of Pediatric Surgery, MosaKids Children’s Hospital, Maastricht University Medical Center+ (MUMC+), Maastricht, the Netherlands

^13^ European Consortium of Pediatric Surgery (Maastricht University Medical Center+, Uniklinik Aachen, Centre Hospitalier Chrétien Liège)

^14^ Department of Epidemiology and Data Science, and the Amsterdam Public Health research institute, Amsterdam UMC, the Netherlands.

^15^ Department of Pediatric Surgery, Juliana Children’s Hospital, Haga Hospital, Den Haag, the Netherlands

**Corresponding author.** L.E. de Vreeze, M.D., [l.e.devreeze@amsterdamumc.nl](mailto:l.e.devreeze@amsterdamumc.nl), Amsterdam UMC location AMC, Meibergdreef 9 1105 AZ Amsterdam, The Netherlands **ORCID ID**; 0009-0005-1466-9631

**Supplementary Materials - Index**

| **Supplementary Methods** |  |
| --- | --- |
| Trial design and sample | *page 3* |
| Patient reported outcome measures (PROMs) | *page 3* |
| Outcomes | *page 3-4* |
| Statistical analysis | *page 4* |
| Ethical Approval | *Page 5* |
| **Supplementary Results** |  |
| 1 year follow-up | *page 6* |
| **Supplementary Figures and Tables** |  |
| Supplementary table 1. Overview of patient characteristic of total cohort and between responders versus non-responders | *page 7* |
| Supplementary table 2. Changes in reported symptoms before and after hernia repair at one year follow-up | *page 8* |
| Supplementary table 3. Uni and multivariable logistic regression analysis for persistent pain and discomfort 4 weeks postoperatively | *page 9* |
| **References** | *page 10* |

**Supplementary Methods**

*Trial design and sample*

This study is a plannend secondary analysis of data from the multicentre randomized controlled trial, titled “The Hernia Exploration oR Not In Infants Analysis (HERNIIA)”, conducted at eight sites in the Netherlands and Belgium between April 2019 and May 2023. In this trial, 402 infants aged 0-6 months with a primary unilateral inguinal hernia scheduled for open hernia repair were randomized into groups 1) with contralateral inguinal exploration (CE) to identify a **contralateral patent processus vaginalis (CPPV)** and 2) no CE. **If a CPPV was identified, contralateral inguinal hernia repair was performed during the same procedure. HERNIIA-trial follow-up was conducted 4 weeks and 1 year after surgery. Main results showed that** additional CE results in fewer re-operations compared with unilateral repair alone however, the number needed to treat (NNT) is high (n = 24); therefore, CE should not be implemented as standard care.

Inclusion criteria were infants aged 0-6 months with a primary unilateral inguinal hernia undergoing open hernia repair. Exclusion criteria included 1) incarcerated inguinal hernia requiring emergency surgery, 2) a ventricular-peritoneal drain and 3) non-descended testis. As is customary in RCT’s, the sample size calculation for the HERNIIA-trial was based on the primary outcome, i.e. re-operation between groups. No separate power calculations were performed for secondary outcomes. Full details of the trial design, population, procedures, and statistical analysis plan have been published previously [1]. The trial was registered at ClinicalTrials.gov (NCT03623893).

*Patient reported outcome measures (PROMs)*

All patients’ parents were asked to fill out two digital questionnaires regarding their child’s health state and quality of life; EuroQol-5 Dimensions-5 Levels (EQ-5D-5L) and the TNO-AZL Preschool Children’s Quality of Life questionnaire (TAPQOL) prior to surgery and 4 weeks after surgery. The digital questionnaires were distributed at the time of enrolment (i.e. randomization) via a secure electronic system that directly sent the questionnaire link to the parents’ e-mail address, and the completion date of the digital questionnaire could be extracted.

*Outcomes*

Primary outcome of this secondary analysis of the HERNIIA-trial was the presence of pain and discomfort prior to surgery and 4 weeks after surgery, assessed using a single-item question (dimension) of the proxy version of EQ-5D-5L. Secondary outcomes included the presence of colic (abdominal cramps) and stomach aches at 4 weeks postoperatively. The latter two outcomes were assessed using two single questions regarding abdominal problems of the TAPQOL questionnaire (stomach domain). The EQ-5D-5L and TAPQOL questionnaires used Likert scales of 5-points (no, slight, moderate, severe and extreme) and 3-points (never, sometimes and often) respectively. For analysis, responses were dichotomized into ‘symptoms present’ (ranging from slight to extreme for the EQ-5D-5L and, sometimes to often for the TAPQOL) and ‘no symptoms’ (no or never). In this secondary analysis, the selected PROM items were used to quantify the frequency of specific, clinically observable symptoms rather than to calculate composite HRQoL domain scores. The pain/discomfort dimension of the EQ-5D and the stomach problems items of the TAPQOL were analysed individually in accordance with their conceptual design and intended reporting applications. Data on obstipation and laxative use were collected through file data extraction from outpatient clinic documentation and follow-up telephone consultations at week 4 postoperatively. Persistent pain or discomfort was defined as symptoms still present at 4 weeks after surgery, independent of the severity.

*Statistical analysis*

Statistical analysis was conducted using IBM SPSS Version 28.0. Descriptive statistics were reported as proportions and percentages for binary or categorical variables and as mean with standard deviation (SD) or as median with interquartile range (IQR) for continuous variables. To assess changes in our dichotomous variables (e.g. presence of pain and discomfort) between paired observations (e.g. preoperative and postoperative), the McNemar’s test was applied. Paired analyses were conducted using available-case data, including only patients with complete data at the respective time points. For our primary outcome, we performed a multivariable logistic regression analysis to identify possible risk factors for persistent complaints of pain and discomfort 4 weeks after surgery. Given there is no available literature on predictors of persistent postoperative symptoms in this population, input variables for the multivariable model were selected based on expert opinion and clinical relevance. Preference was given to factors known preoperatively and considered possibily associated with pain or discomfort in the context of inguinal hernia. Input variables included birth status, sex, history of incarceration, side of the inguinal hernia, and the presence of hernial sac contents observed during surgery. Backward Wald selection was applied for the selection of variables using the standard p=0.10 for variable removal. Effect estimates were reported as odds ratios (OR) with 95% confidence intervals (95%-CI). A p-value <0.05 was considered statistically significant.

*Ethical Approval*

Ethical approval for this study was obtained from the Medical Ethics Committee of Amsterdam University Medical Centre (registration number: 2017.596) and endorsed by the local ethics boards of all participating centres. Written informed consent was obtained from all parents or legal guardians.

The trial was conducted in accordance with Good Clinical Practice guidelines and the principles of the Declaration of Helsinki [2]. The sponsor of this trial (the Netherlands Organization for Health Research and Development, ZonMw) had no role in its design, site selection and collection, analysis, monitoring and interpretation of data and in writing of the manuscript.

**Supplementary Results**

*1 year follow-up*

For all outcomes (pain and discomfort, colic, stomach ache, obstipation and laxative use), the same methodological approach was applied to evaluate changes in symptoms between baseline and at 1-year follow-up. All declining trends continued up to 1 year after hernia repair with symptom frequencies remaining significantly lower compared to baseline. Results are shown in Table S2.

**Supplementary Figures and Tables**

| **Table S1.** Overview of patient characteristic of total cohort and between responders versus non-responders | | | | | | | | | | |
| --- | --- | --- | --- | --- | --- | --- | --- | --- | --- | --- |
|  | Total | Preoperatively | |  |  | Postoperatively | |  |  |  |
|  | (n=402) | | Responders  (n=240) | Non-Responders (n=162) | *p* | Responders  (n=269) | Non-Responders  (n=133) | | *p* |  |
| **Gender** *(male)* | 341 (84.8) | | 204 (85) | 137 (84.6) | 0.906 | 227 (84.4) | 114 (85.7) | | 0.727 |  |
| **Gestational age** *(weeks)* | 37.3 (34.3 – 39) | | 37 (34-39) | 37 (33-39) | 0.536 | 37 (34-39) | 37 (33-39) | | 0.912 |  |
| **Age at surgery** *(weeks)* | 12 (9 – 16) | | 12 (9-15) | 13 (9-18) | 0.222 | 12 (9-16) | 13 (9-18) | | 0.081 |  |
| **Birth status** |  | |  |  | 0.379 |  |  | | 0.248 |  |
| Preterm | 173 (43.0) | | 99 (41.3) | 74 (45.7) | - | 119 (44.2) | 54 (40.6) | | - |  |
| Term | 229 (57.0) | | 141 (58.8) | 88 (54.3) | - | 150 (55.8) | 79 (59.4) | | - |  |
| **Birthweight** *(grams)* | 2710 (1990-3270) | | 2720 (2000-3276) | 2695 (1882-3239) | 0.322 | 2682 (1997-3265) | 2800 (1882-3300) | | 0.891 |  |
| **Side** *(right)* | 252 (62.7) | | 154 (64.2) | 98 (60.5) | 0.455 | 170 (63.2) | 82 (61.7) | | 0.763 |  |
| **Days between presentation and surgery** |  | | 17 (12-29) | 20 (11-35) | 0.230 | 18 (12-29) | 20 (11-35) | | 0.439 |  |
| **History of hernia incarceration** *(yes)* | 54 (13.4) | | 30 (12.5) | 24 (14.8) | 0.520 | 37 (13.8) | 17 (12.8) | | 0.777 |  |
| **ASA** |  | |  |  | 0.725 |  |  | | 0.253 |  |
| I | 215 (53.5) | | 133 (55.4) | 82 (50.6) | - | 151 (56.1) | 64 (48.1 | | - |  |
| II | 124 (30.8) | | 73 (30.4) | 51 (31.5) | - | 83 (30.9) | 41 (30.8) | | - |  |
| III | 29 (7.2) | | 16 (6.7) | 13 (8.0) | - | 16 (5.9) | 13 (9.8) | | - |  |
| Missing | 43 (8.5) | | 18 (7.5) | 16 (9.9) | - | 19 (7.1) | 15 (11.3) | | - |  |
| **Content hernial sac during surgery** *(yes)* | 58 (14.4) | | 35 (14.6) | 23 (14.2) | 0.940 | 38 (14.1) | 20 (15) | | 0.772 |  |
| **Treatment group HERNIIA-trial** |  | |  |  | 0.872 |  |  | | 0.720 |  |
| No CE | 204 (50.7) | | 119 (49.6) | 79 (48.8) | - | 124 (46.1) | 74 (55.6) | |  |  |
| With CE | 198 (49.3) | | 121 (50.4) | 83 (51.2) | - | 145 (53.9) | 59 (44.4) | |  |  |
| *Note. Data are presented as n (%) or median (IQR). Categorical variables were compared using the χ² test. Continuous variables were compared using the Mann–Whitney U test. A p-value <0.05 was considered statistically significant.* | | | | | | | | |  | |

| **Table S2.** Changes in reported symptoms before and after hernia repair at one year follow-up | | | |
| --- | --- | --- | --- |
|  | **Prior to surgery** | **1 year**  **after surgery** |  |
|  | *n/n (%)* | *n/n (%)* | *p†* |
| Pain and discomfort | 125 / 178  (70.2) | 31 / 178  (17.4) | **<0.001** |
| Stomach ache or abdominal pain | 78 / 182  (42.9) | 53 / 182  (29.1) | **0.005** |
| Colic (abdominal cramps) | 173 / 182  (95.1) | 32 / 182  (17.6) | **<0.001** |
| Obstipation | 68 / 397  (17.1) | 16 / 397  (4.0) | **<0.001** |
| Laxative use | 38 / 399  (9.5) | 16 / 399  (4.0) | **0.003** |
| *Note.*  †McNemar’s test, p-value <0.05 was considered statistically significant | | | |

| **Table S3.** Uni and multivariable logistic regression analysis for persistent pain and discomfort 4 weeks postoperatively. | | |
| --- | --- | --- |
| (**A**) | **OR (95% CI)** | ***p*** |
| Birth status |  |  |
| Term (born >38 weeks gestation) | Ref |  |
| Premature (born <37 weeks gestation) | 0.846 (0.495 – 1.509) | 0.607 |
| Sex |  |  |
| Female | Ref |  |
| Male | 1.229 (0.555 - 2.725) | 0.611 |
| History of hernia incarceration |  |  |
| No | Ref |  |
| Yes | 2.072 (0.996-4.311) | 0.051 |
| Side of inguinal hernia |  |  |
| Left | Ref |  |
| Right | 1.953 (1.052 - 3.627) | 0.034 |
| Content hernial sac during surgery |  |  |
| No content | Ref |  |
| Yes content | 0.420 (0.157 - 1.124) | 0.084 |
|  |  |  |
| (**B**) | | |
| History of hernia incarceration |  |  |
| No | Ref |  |
| Yes | 1.933 (0.917-4.075) | 0.083 |
| Side of inguinal hernia |  |  |
| Left | Ref |  |
| Right | **1.898 (1.014-3.552)** | **0.045** |
| Content hernial sac during surgery |  |  |
| No content | Ref |  |
| Yes content | 0.420 (0.156-1.135) | 0.087 |
| *Note.* (A) Univariable logistic regression for the association between baseline characteristics and pain and discomfort 4 weeks after surgery (B) Multivariable logistic regression for the association between baseline characteristics and pain and discomfort 4 weeks after surgery. Final multivariable model including hernia side, incarceration history, and hernial sac content. Birth status and sex were excluded after univariable analysis (p ≥ 0.10). | | |

**References**

1. Maat SC, Dreuning KMA, Anema HR, van Baren R, Been JV, Bender M, et al. Contralateral exploration during open inguinal hernia repair in infants aged 0-6 months to prevent recurrent hernia surgery: a multicenter randomized controlled trial (HERNIIA trial). Int J Surg 2026;112:3661-3668.
2. World Medical Association. World Medical Association Declaration of Helsinki: ethical principles for medical research involving human subjects. JAMA 2013; 310: 2191–2194.
